# USP7 deubiquitinates and stabilizes NOTCH1 in T-cell acute lymphoblastic leukemia

**DOI:** 10.1038/s41392-018-0028-3

**Published:** 2018-10-26

**Authors:** Huizhuang Shan, Xiangyun Li, Xinhua Xiao, Yuting Dai, Jinyan Huang, Junjun Song, Meng Liu, Li Yang, Hu Lei, Yin Tong, Li Zhou, Hanzhang Xu, Yingli Wu

**Affiliations:** 1 Hongqiao International Institute of Medicine, Shanghai Tongren Hospital/Faculty of Basic Medicine, Chemical Biology Division of Shanghai Universities E-Institutes, Key Laboratory of Cell Differentiation and Apoptosis of the Chinese Ministry of Education, Shanghai Jiao Tong University School of Medicine, Shanghai, 200025 China; 2 Department of Hematology, Rui-Jin Hospital, Shanghai Jiao Tong University School of Medicine, No.197, Ruijin Er Road, Shanghai, China; 3 Shanghai University of Medicine & Health Sciences, No.279, Zhouzhu Road, Shanghai, China; 4 Department of Hematology, Shanghai First People’s Hospital, Shanghai Jiao Tong University School of Medicine, Shanghai, 200080 China

## Abstract

T-cell acute lymphoblastic leukemia (T-ALL) is a highly aggressive leukemia that is primarily caused by aberrant activation of the NOTCH1 signaling pathway. Recent studies have revealed that posttranslational modifications, such as ubiquitination, regulate NOTCH1 stability, activity, and localization. However, the specific deubiquitinase that affects NOTCH1 protein stability remains unestablished. Here, we report that ubiquitin-specific protease 7 (USP7) can stabilize NOTCH1. USP7 deubiquitinated NOTCH1 in vivo and in vitro, whereas knockdown of USP7 increased the ubiquitination of NOTCH1. USP7 interacted with NOTCH1 protein in T-ALL cells, and the MATH and UBL domains of USP7 were responsible for this interaction. Depletion of USP7 significantly suppressed the proliferation of T-ALL cells in vitro and in vivo, accompanied by downregulation of the NOTCH1 protein level. Similarly, pharmacologic inhibition of USP7 led to apoptosis of T-ALL cells. More importantly, we found that USP7 was significantly upregulated in human T-ALL cell lines and patient samples, and a USP7 inhibitor exhibited cell cytotoxicity toward primary T-ALL cells, indicating the clinical relevance of these findings. Overall, our results demonstrate that USP7 is a novel deubiquitinase that stabilizes NOTCH1. Therefore, USP7 may be a promising therapeutic target in the currently incurable T-ALL.

## Cancer pathology: Cutting a lifeline of lymphoblastic leukemia

Inhibiting the protein ubiquitin-specific protease 7 (USP-7) may offer a treatment option for patients with T-cell acute lymphoblastic leukemia (T-ALL), an incurable and aggressive cancer. T-ALL is primarily caused by aberrant activation of the protein NOTCH1, which regulates gene expression relating to cell survival, proliferation, and metastasis. Ying-Li Wu, from China’s Shanghai Jiao Tong University School of Medicine, and a team of researchers report that, in their experiments, USP-7 prevented the breakdown of NOTCH1 by removing the protein tag that would normally signal it for degradation. In addition, USP-7 expression was upregulated in all human T-ALL cells and samples tested. Inhibition of USP-7 suppressed the proliferation of T-ALL cells in vitro and in vivo. These results reveal the role of USP-7 in the pathophysiology of T-ALL and offer USP-7 as a promising potential therapeutic target.

## Introduction

The NOTCH1 receptor is a transmembrane protein that serves as a ligand-activated transcription factor that regulates a great diversity of cellular events, including cell proliferation, survival, metastasis, and differentiation.^1^ Upon ligand binding, NOTCH1 is initially cleaved by an ADAM metalloprotease in tandem with the γ-secretase complex, which releases the intracellular domain of NOTCH1 (ICN1). Then, ICN1 translocates into the nucleus and activates NOTCH1 target genes, such as *c-Myc, Hes1*, and *Dtx1*.^2–5^ Activating mutations in *NOTCH1* that induce ligand-independent activation of the receptor or an increase in the stability of ICN1 are found in more than 60% of human T-cell acute lymphoblastic leukemia (T-ALL) cases. T-ALL is one of the most aggressive leukemias and has a poor prognosis.^6–11^ A tremendous amount of research has focused on the oncogenic mechanisms by which NOTCH1 enhances leukemogenesis via downstream genes or interaction with other important signaling pathways, such as NF-κB and PI3K-AKT-mTOR pathways.^12,13^ However, the upstream mechanisms sustaining aberrant NOTCH1 signaling activities are incompletely understood, especially NOTCH1 protein turnover.

It is known that the ubiquitin-proteasome system and lysosome pathway participate in the regulation of NOTCH1 turnover. For instance, the E3 ubiquitin ligases F-box and WD repeat domain-containing 7 (FBW7) and C-terminus of Hsc70-interacting protein (CHIP) mediate polyubiquitination of NOTCH1 for proteasome degradation.^14,15^ NOTCH1 interacts with and is monoubiquitinated by the E3 ubiquitin ligase c-Cbl and is subsequently degraded by lysosomes.^16^ Ubiquitination is a reversible process, and removal of ubiquitin from proteins is mediated by deubiquitinases (DUBs), the number of which in mammalian cells is ~100. More than the half of DUBs belong to the ubiquitin-specific protease (USP) subfamily.^17^ To date, eIF3f has been reported to function as a deubiquitinase and to regulate the activation of NOTCH1.^18^ However, the deubiquitinase that modulates the stability of NOTCH1 protein remains unknown.

USP7 is the most widely studied DUB and is well known as herpes-associated USP (HAUSP).^19^ Through its deubiquitination activity, USP7 can influence the localization, activation, and stability of its substrates. For example, USP7 changes the localization of monoubiquitinated FOXO4 and PTEN through removal of the single ubiquitin molecule^20–22^ and can regulate the stability of p53, MDM2, N-MYC, TRIP12, FOXP3, ASXL1, UHRF1, PHF8, and DNMT1.^23–30^ Many of the preceding factors are critical in cancer development, epigenetic control, cell signaling, DNA damage repair, and immune responses. Notably, overexpression of USP7 has been detected in multiple myeloma, neuroblastoma, hepatocellular carcinoma, prostate cancer, breast cancer, and ovarian cancer, in which inhibition of USP7 suppresses proliferation and induces death of cancer cells independently of their p53 status. Considering the crucial role of USP7 in cancer development, much attention has been paid to developing USP7 inhibitors for cancer therapy.^31–35^

In this study, we confirmed that USP7 is a novel deubiquitinase that reverses NOTCH1 polyubiquitination and stabilizes NOTCH1 protein. Inhibition of USP7 led to NOTCH1 degradation and suppressed T-ALL cell proliferation in vitro and in vivo. Our data suggest that targeting the USP7/NOTCH1 axis is a novel strategy to combat T-ALL and other NOTCH1-related malignancies.

## Materials and methods

### Cell culture, patient samples, and transfection

The human T-ALL cell lines JURKAT and MOLT-4 and human embryonic kidney (HEK293T) cells were purchased from the American Type Culture Collection (ATCC, Manassas, VA, USA). CUTLL1 cells were a gift from Dr. Qingyi Tong (Huazhong University of Science and Technology, Wuhan, China); CCRF-CEM, KOPT-K1, SIL-ALL, HPB-ALL, DND41, and LOUCY cell lines were kindly provided by Dr. Xinhua Xiao (Shanghai Jiao Tong University School of Medicine, Shanghai, China). T-ALL cell lines were cultured in RPMI-1640 medium with 2 mM l-glutamine (Gibco Invitrogen Corp., Grand Island, NY, USA) supplemented with 10% fetal bovine serum (FBS; Gibco) and 1% penicillin/streptomycin (Gibco). HEK293T cells were cultured in Dulbecco’s modified Eagle’s medium (DMEM; HyClone, Logan, UT, USA) containing 10% FBS and 1% penicillin/streptomycin. Peripheral blood mononuclear cells (PBMCs) were isolated from normal healthy donors or T-ALL patient samples provided by the Department of Hematology, Rui-Jin Hospital, Shanghai Jiao Tong University School of Medicine, Shanghai, China. Studies were carried out in accordance with guidelines approved by the Clinical Investigational Reviewing Board of the Shanghai Jiao Tong University School of Medicine. The cells listed above were maintained at 37 °C in a humidified incubator with 95% air and 5% CO_2_. HEK293T cells were transfected with plasmids using polyethyleneimine (PEI; Polysciences, Warrington, PA, USA) according to the manufacturer’s instructions.

### Plasmids, antibodies, and reagents

DUB plasmids and HA-ubiquitin were purchased from Addgene (Cambridge, MA, USA). USP7^WT^ (USP7 wild-type) and USP7^C223S^ (USP7 catalytic mutant) were cloned into a pFLAG-CMV-4 vector. GFP-tagged USP7 constructs (WT or mutants) were kindly provided by Prof. Jing Liu (Central South University, Changsha, China). Additionally, pcDNA3-Myc-ICN1 and pcDNA3-FLAG-ICN1 were obtained from Prof. Hudan Liu (Wuhan University, Wuhan, China).

The following antibodies were used in this study: anti-cleaved NOTCH1 (Val1744) and anti-β-Actin (CST, Danves, MA, USA); anti-USP7 (Bethyl Laboratories, Montgomery, TX, USA); anti-NOTCH1 and anti-GFP (Santa Cruz, Dallas, TX, USA); anti-HA and anti-Myc epitope tag (MBL, Nagoya, Japan); anti-FLAG (M2) (Sigma-Aldrich, Louis, MO, USA); and anti-HRP-conjugated secondary antibody (Millipore, Bedford, MA, USA). All antibodies were diluted according to the manufacturer’s recommendations.

The USP7 inhibitor P22077 [1-(5-((2,4-difluorophenyl)thio)-4-nitrothiophen-2-yl) ethanone] was obtained from EMD Millipore (EMD Millipore, Billerica, MA, USA). MG132 and cycloheximide (CHX) were purchased from Sigma-Aldrich.

### Retroviral transduction and infection

shRNA sequences were inserted into a PSIREN-RetroQ Vector (Clontech Laboratories, Inc., CA, USA), and then, HEK293T cells were co-transfected with the construct and packaging plasmids (pGag-Pol and pVSV-g). At 48 h posttransfection, the supernatants were collected and filtered using a 0.45-μm cellulose acetate filter. Further, the JURKAT and MOLT-4 cells were incubated in culture medium with supernatants containing virus particles and supplemented with 8 μg/mL polybrene (Sigma-Aldrich) for 8 h, followed by replacement with fresh medium. At 48 h postinfection, puromycin (2 μg/mL; Calbiochem, Merck KGaA, Darmstadt, Germany) was added to screen the cells. Control shRNA was synthesized by Sangon Biotech (Shanghai, China), and the following USP7 shRNA-1 and USP7 shRNA-2 sequences were used: USP7 shRNA-1, 5′-TGCGAAATCTGCCATGGAA-3′; USP7 shRNA-2, 5′-CTCAGAACCCTGTGATCAA-3′.

### RNA extraction and quantitative real-time PCR

Total RNA was extracted with TRIzol reagent (Invitrogen), and cDNA was synthesized using a reverse transcriptase kit (Thermo Scientific, Waltham, MA, USA), followed by qRT-PCR analysis using SYBR-Green qPCR master mix (Thermo Scientific) and an ABI PRISM 7900 system (Thermo Scientific). The primer sequences were as follows: NOTCH1 forward 5′-CCGCAGTTGTGCTCCTGAA-3′ and NOTCH1 reverse 5′-ACCTTGGCGGTCTCGTAGCT-3′; β-actin forward 5′-CATCCTCACCCTGAAGTACCC-3′ and β-actin reverse 5′-AGCCTGGATAGCAACGTACATG-3′.

### Immunoprecipitation and western blotting

For immunoprecipitation assays, cells were lysed in lysis buffer (1% Nonidet P-40; 50 mM Tris-HCl, pH 7.4; 150 mM NaCl; 5 mM EDTA; 0.02% SDS) supplemented with protease inhibitors (Roche Applied Science) on ice for 30 min. After centrifugation at 4 °C and 12,000 rpm for 20 min, the supernatants were incubated with the indicated primary antibodies at 4 °C overnight. Protein A/G Plus agarose beads (Santa Cruz) were then added for 3 h. Next, the beads were washed with the lysis buffer three times. Finally, the bound proteins were dissolved in 2× SDS-PAGE loading buffer and analyzed by western blotting. For in vivo deubiquitination assays, cells were transfected with the aforementioned plasmids for 48 h. 4 h before the collection of cell lysates, 10 μM MG132 was added. Cell lysates were then subjected to immunoprecipitation experiments as described above.

For western blotting, cells were collected and lysed with 1× SDS-PAGE sample buffer. Approximately 30 μg of total protein extracts was resolved on 8–15% SDS-PAGE gels (8–15%), electrophoresed, and transferred to a nitrocellulose membrane (Bio-Rad, Hercules, CA, USA). To ensure equal protein loading, the blots were stained with 0.2% Ponceau S red. After being blocked with 5% nonfat milk in phosphate-buffered saline (PBS), the membranes were incubated with the antibodies specified above at 4 °C overnight, followed by incubation with an HRP-conjugated secondary antibody for 1 h at room temperature (RT). Finally, the signals were measured by chemiluminescence (ECL, Amersham, Little Chalfont, UK).

### In vitro deubiquitination assay

For the in vitro deubiquitination assay, Myc-tagged ICN1 was co-expressed with HA-tagged ubiquitin in HEK293T cells and purified using an anti-Myc antibody and Protein A/G Plus agarose beads under denaturing conditions (50 mM Tris- HCl, pH 8.0; 50 mM NaCl; 10 mM DTT; 1 mM EDTA and 5% glycerol). Next, ubiquitinated-ICN1 proteins were incubated with purified USP7 protein (SinoBiological Inc., Beijing, China) in deubiquitination buffer at 37 °C for 2 h. This reaction was terminated by boiling in 5× SDS-PAGE sample buffer for 10 min. Then, the samples were resolved on 8% SDS-PAGE gels, followed by western blotting analysis.

### GST pull-down assay

Bacterial-expressed GST and commercially available GST-USP7 were bound to glutathione-Sepharose 4B beads (GE Healthcare, Sunnyvale, CA, USA). FLAG-tagged ICN1 was expressed in HEK293T cells. Cells were lysed and immunoprecipitated with anti-FLAG M2 beads (Sigma-Aldrich), and then, purified ICN1 protein was eluted using a 3 × FLAG peptide (Sigma-Aldrich). FLAG-tagged ICN1 protein was incubated with GST or GST-USP7 protein in buffer (10 mM Tris-HCl, pH 8.0; 100 mM NaCl; 1 mM EDTA) at 4 °C for 4 h. The beads were washed three times and boiled in 2× SDS-PAGE loading buffer, followed by western blotting analysis.

### Analysis of apoptosis via flow cytometry

Apoptosis in T-ALL cell lines was evaluated using Annexin V-APC and propidium iodide (PI; BD Pharmingen, San Jose, USA) staining. Briefly, cells were treated with different concentrations of P22077 for 48 h. The apoptotic cells were then washed with PBS thrice and stained with Annexin V-APC and PI according to the manufacturer’s protocol. Early apoptotic cells (Annexin V-positive, PI-negative) and late apoptotic cells (Annexin V-positive, PI-positive) were then determined by flow cytometry (BD Biosciences, San Diego, CA, USA), and the results were analyzed using FlowJo 7.6 software (Tree Star, Ashland, OR, USA).

### Cell proliferation analysis

Cell proliferation was determined using a Cell Counting Kit-8 assay kit (Dojindo Laboratories, Kumamoto, Japan). T-ALL cells were seeded into 96-well plates (4 **×**10^3^ cells/well) and then treated with different concentrations of P22077. After incubation at 37 °C for a different number of days, 10 μL of CCK-8 reagent was added to each well. Further, incubation for another 4 h was performed, and the optical density (OD) at 450 nm was determined using a Synergy H4 Hybrid Microplate Reader (Synergy H4, Biotek, Winooski, VT, USA).

### Immunofluorescence assay

Cells were fixed with 4% paraformaldehyde, permeabilized with 0.5% Triton X-100 for 15 min at RT, and blocked with 2% bovine serum albumin in PBS for 2 h at RT. The cells were next incubated with the indicated NOTCH1 (dilution 1:50; Santa Cruz) and USP7 (dilution 1:100; Bethyl Laboratories) primary antibodies overnight at 4 °C, followed by incubation with a TRITC-conjugated or FITC-conjugated secondary antibody (dilution 1:200; Invitrogen) for 2 h at RT. The cell nuclei were counterstained with 4,6-diamidino-2-phenylindole (DAPI; Molecular Probes, Eugene, OR, USA). Confocal imaging was performed using a laser confocal microscope (Nikon, Nagoya, Japan).

### Gene expression analysis of USP7 using databases

The USP7 transcript expression was assessed in human cell lines using the Cancer Cell Line Encyclopedia (CCLE).^36^ The “Haferlach Leukemia” and “Andersson Leukemia” datasets from the Oncomine database (http://www.oncomine.org/)^37^ were employed to analyze the differential expression of USP7 in T-ALL and normal marrow or peripheral blood cells.

RNA-seq data of 130 T-ALL samples were downloaded from the Chinese Genotype-Phenotype Archive of Hematology (http://bioinfo.rjh.com.cn/cga/) under accession no. CGAS00000000002. Mutation data were obtained from a supplementary table in ref. ^38^ Samples with USP7 mutation (*n* = 12) were not taken into account in the downstream analysis. RNA-seq data were aligned against the human reference genome hg19 using STAR (Version 2.5.3a).^39^ HTSeq (Version 0.9.1) was used to generate a table of the counts from the STAR output.^40^ All read counts were normalized and applied with variance-stabilizing transformation in the DESeq2 package.^41^

### T-ALL xenografts

Female NOD/SCID/IL2Rγ-null (NSG) mice aged six to 8 weeks were kept under pathogen-free conditions according to the Shanghai Medical Experimental Animal Care guidelines. Human JURKAT cells transduced with retroviruses encoding pSIREN-Control shRNA or pSIREN-USP7 shRNA-2 were intravenously injected into NSG mice (2.5 × 10^6^ cells in 100 μL of PBS). Disease progression was monitored weekly by Wright’s staining of peripheral blood. After 3 weeks, the mice were killed. Tissue samples were fixed in formaldehyde and further processed for hematoxylin and eosin (H&E) staining. The animal protocols were approved by the Institutional Animal Care and Use Committee of Shanghai Jiao Tong University School of Medicine.

### Statistical analysis

All graphs were obtained using GraphPad Prism 5 software (GraphPad Software Inc., La Jolla, CA, USA). The data were obtained from three independent experiments and are presented as the mean ± standard deviation (SD). Student’s *t*-test was used to determine the significance of differences between groups; *p* *<* 0.05 was considered to indicate statistical significance (^*^p *<* 0.05; ^**^p *<* 0.01; ^***^p < 0.001).

## Results

### USP7 maintains ICN1 stability

**Fig. 1:**
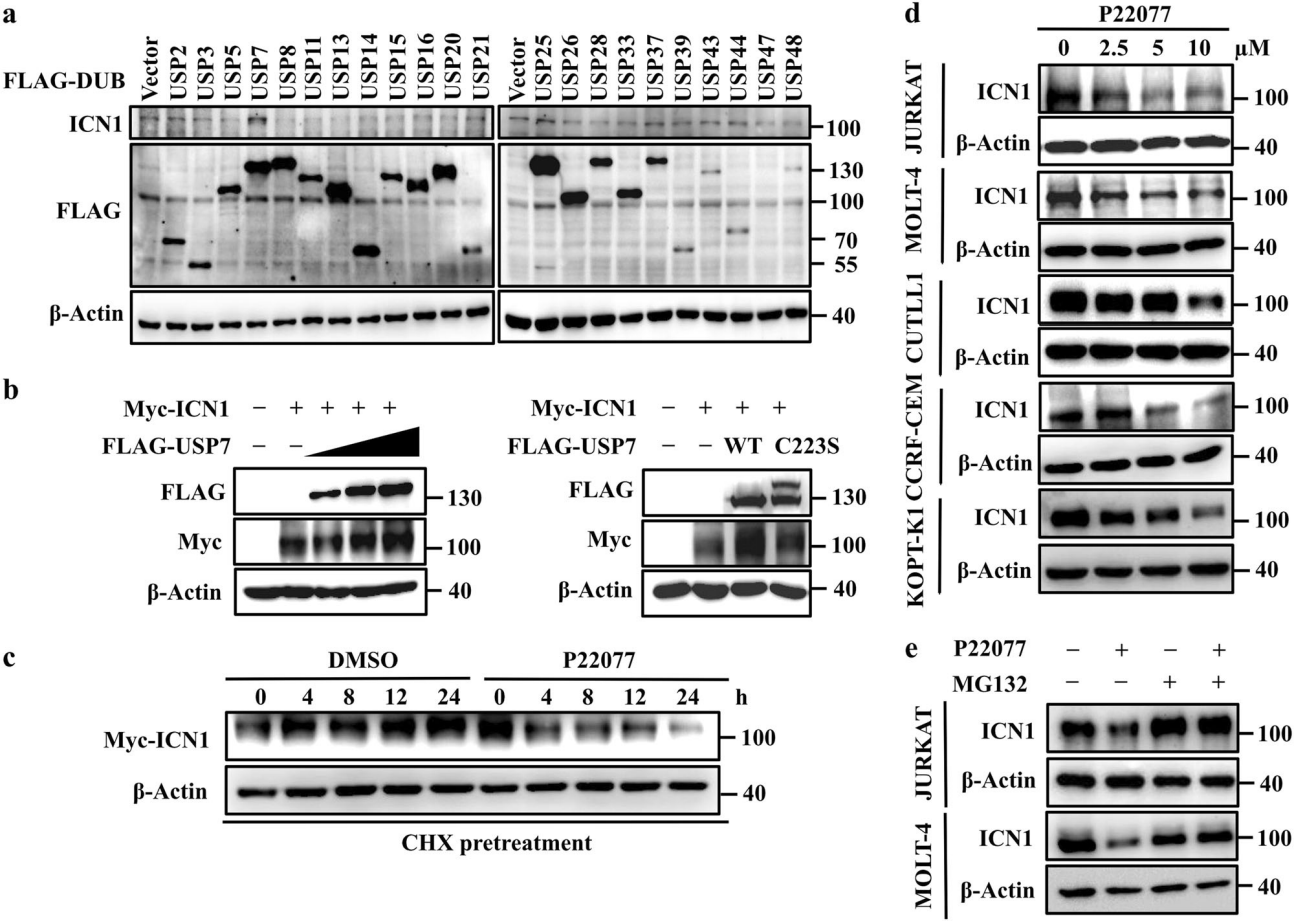
The deubiquitinase USP7 stabilizes ICN1. **a** HEK293T cells were transfected with FLAG-tagged USPs. Cell lysates were subjected to western blotting with anti-FLAG and anti-ICN1 antibodies. **b** HEK293T cells were co-transfected with plasmids encoding Myc-tagged ICN1 and FLAG-tagged USP7 (increasing amounts) or its inactive C223S mutant. Cell lysates were subjected to western blotting with anti-FLAG and anti-Myc antibodies. **c** HEK293T cells were transfected with Myc-tagged ICN1 and pretreated with CHX (100 μg/mL) for 2 h. The cells were harvested at the indicated time points upon DMSO or P22077 (10 μM) treatment followed by western blotting with an anti-Myc antibody. **d** The indicated T-ALL cell lines were treated with increasing concentrations of P22077 for 6 h, and the ICN1 protein levels were measured by western blotting. **e** JURKAT and MOLT-4 cells were treated with P22077 (10 μM) in the presence or absence of MG132 (5 μM) for 6 h; the indicated proteins were then examined by western blotting

USPs are the largest subfamily of DUBs. To explore which DUB is responsible for ICN1 stability, a total of 22 USPs were overexpressed in HEK293T cells, and the protein level of endogenous ICN1 was analyzed. This screening revealed that USP7 dramatically upregulated ICN1 levels (Fig. 1a). Then, to confirm this result, HEK293T cells were co-transfected with ICN1 and different doses of USP7 (USP7^WT^) plasmids, and as expected, the overexpression of USP7 remarkably increased ICN1 levels in a dose-dependent manner (Fig. 1b, left panel). Moreover, regulation of the ICN1 protein level by USP7 was dependent on its DUB activity, as shown by the inability of a catalytically inactive USP7 mutant (USP7^C223S^) to upregulate ICN1 (Fig. 1b, right panel). Consistent with the above results, P22077,^42^ a USP7 inhibitor, significantly shortened the half-life of exogenous ICN1 in HEK293T cells (Fig. 1c).

Considering that NOTCH1 plays a critical role in T-ALL, we next examined how inhibition of USP7 would influence NOTCH1. Treatment of T-ALL cell lines (JURKAT, MOLT-4, CUTLL1, CCRF-CEM, and KOPT-K1) with P22077 led to a reduction in the ICN1 protein level in a dose-dependent manner (Fig. 1d) without changing the *NOTCH1* mRNA level (Supplementary Fig. S1A and S1B). On the other hand, the proteasome-specific inhibitor MG132 blocked the P22077-induced ICN1 downregulation in JURKAT and MOLT-4 cells (Fig. 1e). These results indicate that the deubiquitinase USP7 controls the stability of NOTCH1.

### USP7 interacts with ICN1

**Fig. 2:**
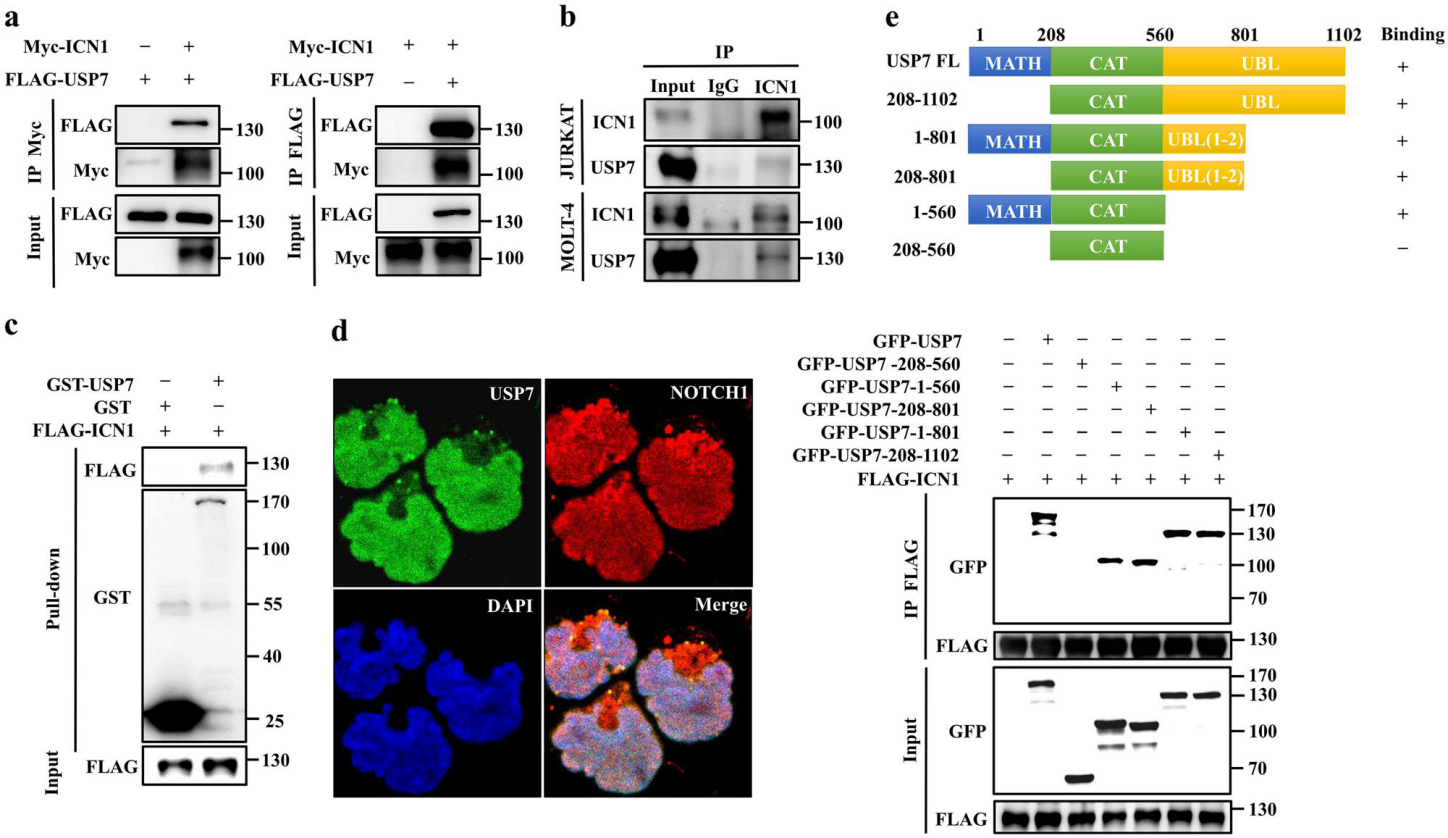
USP7 interacts with ICN1. **a** HEK293T cells were transfected with plasmids encoding FLAG-tagged USP7 and/or Myc-tagged ICN1. Cell extracts were prepared and immunoprecipitated with anti-FLAG or anti-Myc antibodies. The protein interactions were analyzed by western blotting. **b** Whole-cell lysates from JURKAT and MOLT-4 cells were subjected to immunoprecipitation with a control IgG or an anti-ICN1 antibody. The immunoprecipitates were detected by western blotting. The input represented ~5% of the total protein extract used for immunoprecipitation. **c** The direct interaction between USP7 and ICN1 was detected using a GST pull-down assay, and the indicated proteins were examined by western blotting. **d** USP7 was co-localized with NOTCH1. CUTLL1 cells were fixed and immunostained with anti-USP7 (green) and anti-NOTCH1 (red) antibodies. The cell nuclei were counterstained with DAPI (blue). **e** Mapping of the ICN1-interacting domain in the USP7 protein. Top panel, a schematic representation of various USP7 truncated mutants. Bottom panel, HEK293T cells were co-transfected with constructs encoding FLAG-tagged ICN1 and GFP-tagged USP7 or truncated mutants. FLAG-tagged ICN1 proteins were immunoprecipitated with an anti-FLAG antibody, and the presence of USP7 protein and truncated mutants was examined by western blotting using an anti-GFP antibody

Next, we explored whether USP7 interacts with ICN1 using an immunoprecipitation assay and found that ectopically expressed Myc-ICN1 interacted with FLAG-USP7 (Fig. 2a). An interaction between endogenous USP7 and ICN1 was also validated in JURKAT and MOLT-4 cells (Fig. 2b). To investigate whether USP7 interacts directly with ICN1, we carried out GST pull-down assays. As shown in Fig. 2c, purified GST-USP7 but not GST alone could bind to FLAG-ICN1, indicating a direct interaction between USP7 and ICN1. Furthermore, the results of an immunofluorescence assay showed that USP7 and NOTCH1 were co-localized in the nucleus (Fig. 2d).

To determine the precise region of USP7 essential for its interaction with ICN1, we generated several truncated mutants of USP7 (Fig. 2e, top panel). The USP7 protein contains an N-terminal meprin, TRAF homology (MATH) domain (aa 1–208); a single catalytic domain (aa 208–560); and five C-terminal ubiquitin-like (UBL) domains (aa 560–1102). The results revealed that USP7 interacts with ICN1 through its N-terminal MATH domain and C-terminal UBL domains (Fig. 2e, bottom panel), and the UBL (1–2) domain was sufficient to bind ICN1. Nevertheless, the catalytic domain of USP7 itself could not bind ICN1 (Fig. 2e, bottom panel). These data demonstrate that USP7 interacts with ICN1 in vivo and in vitro and the MATH domain and UBL domains of USP7 mediate this interaction.

### USP7 deubiquitinates ICN1

**Fig. 3:**
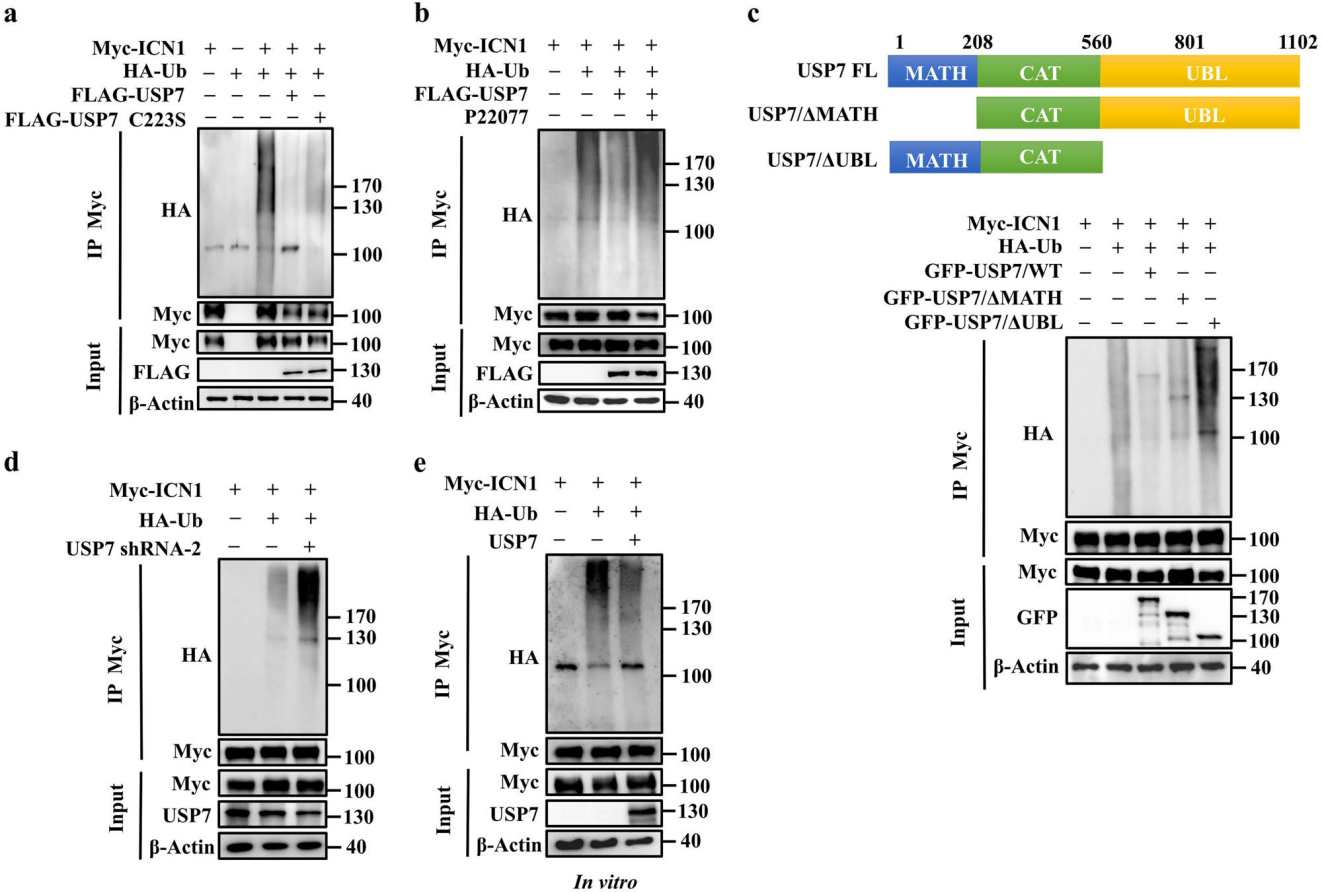
USP7 deubiquitinates ICN1 in vivo and in vitro. **a** HEK293T cells were co-transfected with the specified plasmids. Next, cellular extracts were prepared for immunoprecipitation assays with anti-Myc antibody followed by western blotting with anti-HA antibody. **b** HEK293T cells were co-transfected with the indicated plasmids and treated with or without P22077 (20 μM) for 6 h before being harvested. Further, cellular extracts were prepared for immunoprecipitation assays with anti-Myc antibody followed by western blotting with anti-HA antibody. **c** HEK293T cells were co-transfected with the indicated plasmids. Then, cellular extracts were immunoprecipitated with anti-Myc antibody followed by western blotting with anti-HA antibody. **d** HEK293T cells stably expressing shRNA specifically against USP7 were co-transfected with the indicated plasmids. Cellular extracts were immunoprecipitated with anti-Myc antibody followed by western blotting with anti-HA antibody. **e** In vitro deubiquitination assays. HEK293T cells were co-transfected with HA-tagged ubiquitin and Myc-tagged ICN1. Ubiquitinated-ICN1 proteins were purified using an anti-Myc antibody and then incubated with purified USP7 at 37 °C for 2 h, followed by western blotting with anti-HA antibody

Further, we sought to determine whether USP7, as a deubiquitinase, catalyzes the deubiquitination of ICN1. For this purpose, we prepared HEK293T cells, which were transfected with expression plasmids encoding HA-ubiquitin and Myc-ICN1 with or without USP7^WT^ or USP7^C223S^. Ubiquitinated-ICN1 was immunoprecipitated with an anti-Myc antibody and detected by western blotting with anti-HA antibody. As expected, co-expression of ICN1 with wild-type USP7 but not the catalytically inactive USP7^C223S^ decreased the protein levels of ubiquitinated ICN1 (Fig. 3a). Consistent with this finding, inhibition of USP7 by P22077 completely blocked the ability of USP7 to remove ubiquitin from ICN1 (Fig. 3b). Of note, USP7 mutants lacking the MATH domain or UBL domains could not remove ubiquitin from ICN1 (Fig. 3c), confirming the essential role of the MATH domain and UBL domains in mediating the deubiquitination activity of USP7 for ICN1. Moreover, HEK293T cells stably expressing shRNA specifically against USP7 were co-transfected with plasmids encoding HA-ubiquitin and Myc-ICN1. The protein levels of ubiquitinated ICN1 species increased after the knockdown of USP7 (Fig. 3d). To further confirm our hypothesis that USP7 is a direct DUB for ICN1, we performed an in vitro deubiquitination assay. Ubiquitinated ICN1 proteins were purified from HEK293T cells and incubated with commercially available purified USP7 protein. As illustrated in Fig. 3e, the purified USP7 markedly reduced the ubiquitination of ICN1. Taken together, the results of these experiments indicate that USP7 targets ICN1 for deubiquitination, supporting the proposal that ICN1 is a direct substrate for the deubiquitinase USP7.

### USP7 is overexpressed in T-ALL

**Fig. 4:**
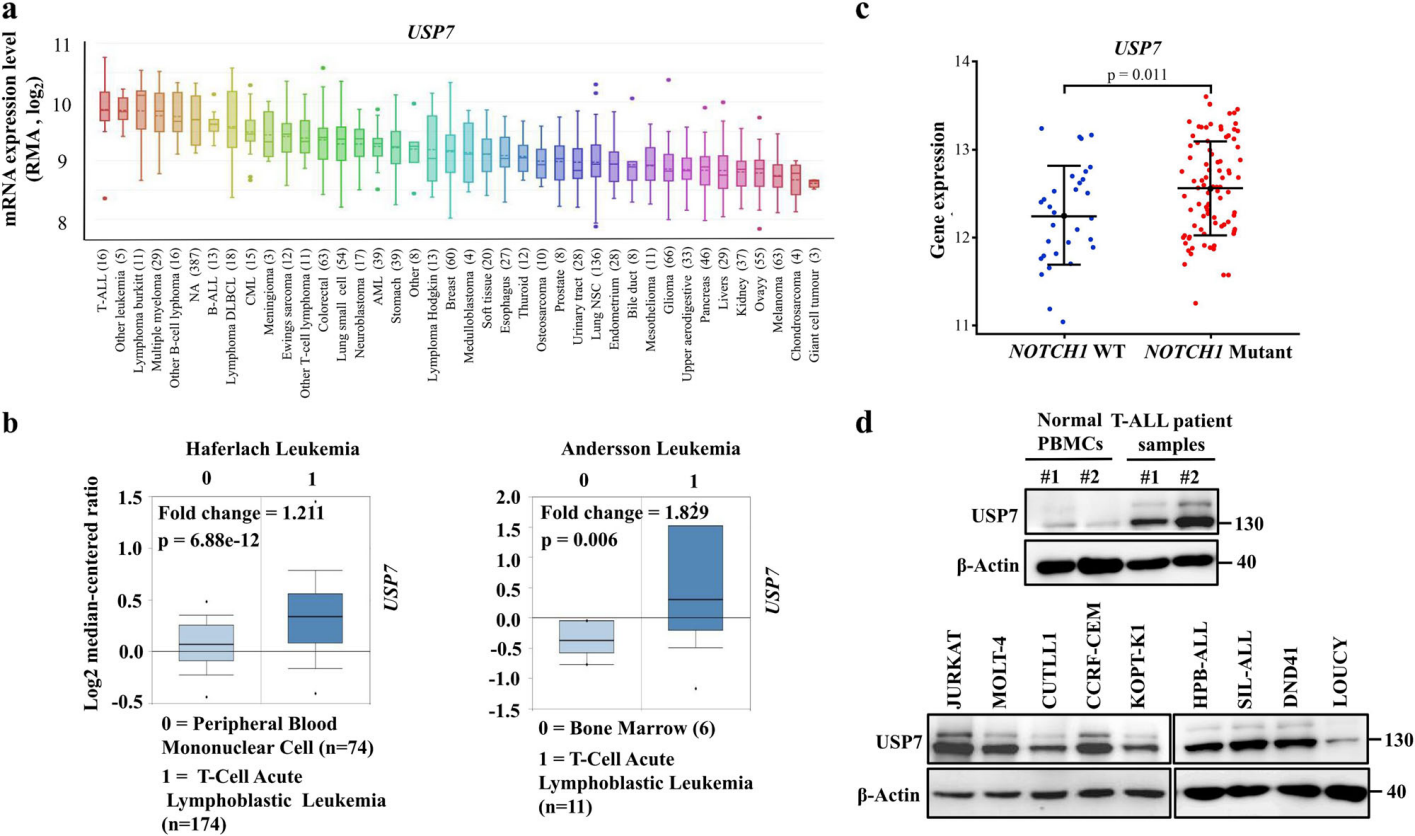
USP7 is overexpressed in T-ALL. **a** USP7 microarray gene expression data were obtained from the Cancer Cell Line Encyclopedia (CCLE). The data are presented with box plots. The sample number (*n*) are indicated in parentheses. RMA represents Robust Multi-array Average. **b** Analysis of TCGA leukemia dataset from the Oncomine database to assess the expression of *USP7* in normal bone marrow or peripheral blood cells and in T-ALL patient samples. The data are presented with box plots. Fold change, *p-*value (determined by Student’s *t*-test), and sample size are shown. **c** Comparison of USP7 gene expression levels between *NOTCH1* WT (*n* = 31) and *NOTCH1* mutated (*n* = 87) T-ALL cases. The read counts mapped to the USP7 transcript were normalized and applied with variance-stabilizing transformation. The *p-*value was determined using Student’s *t*-test. Dots represent the value of the *USP7* expression level in each of the T-ALL cases. The mean and 25th and 75th percentiles are represented by the midline and line edges in the plots, respectively. **d** western blotting analysis of the USP7 protein levels in normal PBMCs and T-ALL patient samples (top panel) along with various T-ALL cell lines (bottom panel)

To assess the USP7 expression in T-ALL, we first analyzed the transcript expression in a wide array of human cancer cell lines and discovered that *USP7* was highly expressed in T-ALL (Fig. 4a). To extend this observation, we carried out bioinformatics analysis using TCGA leukemia datasets from the public database Oncomine. *USP7* was markedly upregulated in T-ALL samples compared with its expression in normal bone marrow or peripheral blood cells (Fig. 4b). Interestingly, the expression level of *USP7* was considerably higher in patients suffering from *NOTCH1* mutant T-ALL than in those with wild-type *NOTCH1* (Fig. 4c). Additionally, USP7 was highly expressed in both human T-ALL cell lines (Fig. 4d, bottom panel) and primary T-ALL patient samples (*n* = 2; Fig. 4d, top panel). Overall, these observations indicate that USP7 tends to be expressed at a higher level in human T-ALL.

### Knockdown of USP7 reduces ICN1 protein levels and suppresses T-ALL cell proliferation in vitro and in vivo

**Fig. 5:**
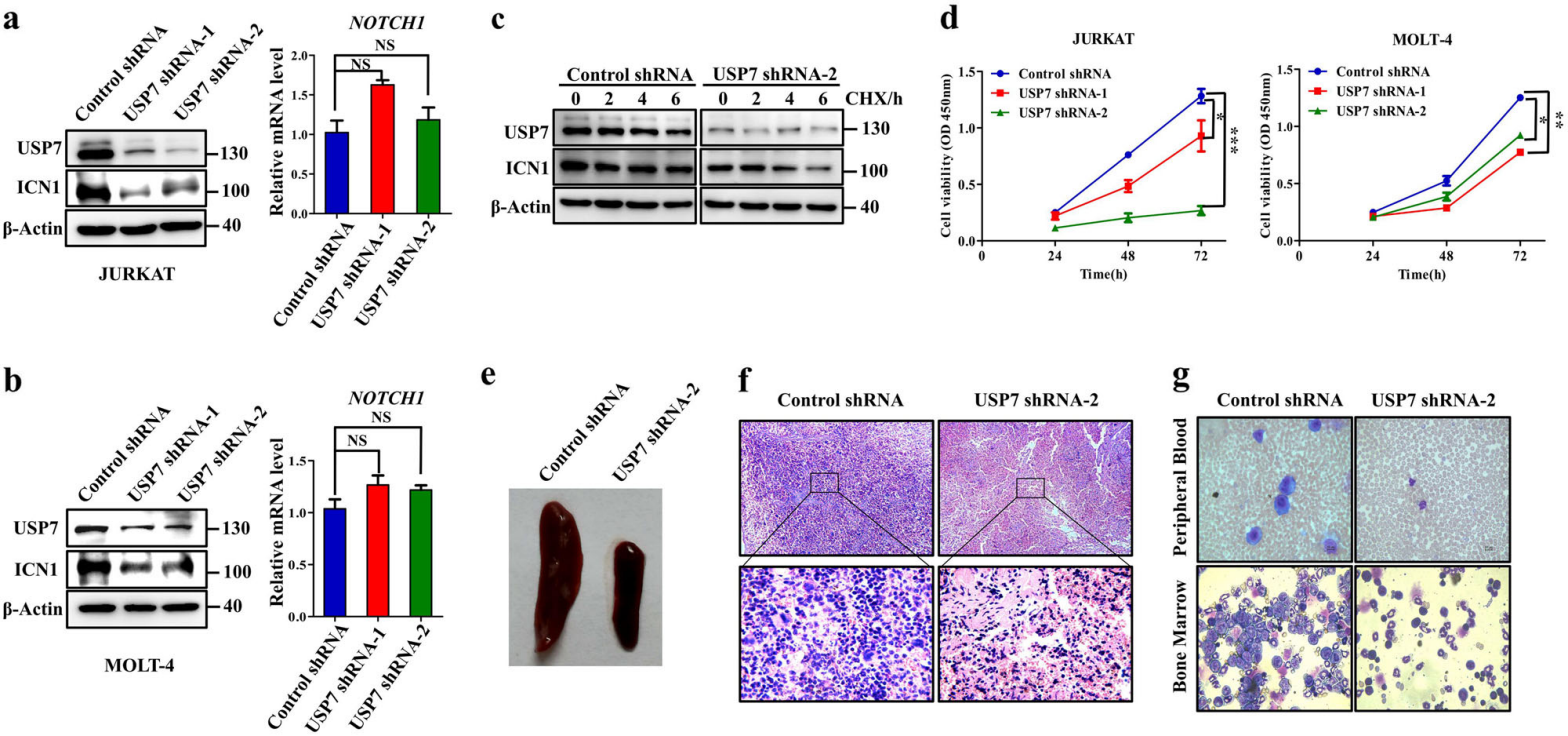
Knockdown of USP7 reduces ICN1 protein levels and suppresses T-ALL cell proliferation in vitro and in vivo. **a**, **b** Knockdown of USP7 reduces ICN1 protein levels. JURKAT (**a**) and MOLT-4 cells (**b**) were infected with control shRNA or two different shRNAs against USP7. The protein expression levels of USP7 and ICN1 were detected using western blotting (left panel), and the mRNA levels were determined by qRT-PCR (right panel). The data are presented as the mean ± S.D. (Student’s *t*-test) of three independent experiments; NS denotes no significant. **c** JURKAT cells transfected with control shRNA or USP7 shRNAs were treated with CHX (50 μg/mL) and harvested at the indicated time points, followed by western blotting analysis. **d** JURKAT and MOLT-4 cells were infected with control shRNA or USP7 shRNAs, and cell proliferation was monitored by a CCK-8 assay. The data are presented as the mean ± S.D. (Student’s *t*-test) of three independent experiments; ^*^p *<* 0.05, ^**^p *<* 0.01, and ^***^p < 0.001. **e** JURKAT cells stably expressing the control shRNA or USP7 shRNAs were intravenously injected into NSG mice (*n* = 3 per group). After 3 weeks, the mice were killed, and their T-ALL progression was analyzed. Representative spleen images are shown. **f** Representative spleen H&E staining is presented (top, 200×; bottom, 1000×). **g** Wright’s staining of peripheral blood (top panel) and bone marrow (bottom panel) cytospin samples from the control mice and USP7-depleted mice

Since NOTCH1 plays a significant role in T-ALL pathogenesis, we proposed that loss of function of USP7, which deubiquitinates and stabilizes ICN1, might inhibit T-ALL cell growth. As depicted in Fig. 5a, b (left panel), knockdown of USP7 by two individual shRNAs significantly reduced the endogenous ICN1 protein levels in JURKAT and MOLT-4 cells without affecting the *NOTCH1* mRNA level (Fig. 5a, b, right panel). Western blotting analysis revealed that USP7 depletion was associated with a decreased ICN1 half-life (Fig. 5c). Moreover, the knockdown of USP7 significantly suppressed the proliferation of JURKAT and MOLT-4 cells (Fig. 5d).

To further confirm these observations in vivo, 2.5 million JURKAT cells with or without USP7 expression were intravenously injected into NOD/SCID/IL2Rγ-null (NSG) mice. At 23 days postengraftment, the control cohort showed typical leukemia phenotypes and began to die. Notably, the spleens of the USP7-depleted cohort were much smaller than those of the control cohort (Fig. 5e). Hematoxylin and eosin (H&E) staining indicated that the USP7-depleted mice had less infiltration of lymphoblastic leukemia cells into the spleen in comparison with the control mice (Fig. 5f). Moreover, Wright’s staining analysis revealed massive elevation of the number of human leukemia cells in the peripheral blood and bone marrow from the control mice. In contrast, much fewer human leukemia cells were detected in the USP7-depleted mice (Fig. 5g). These studies provide compelling evidence supporting the critical importance of USP7 as an upstream mediator of NOTCH1 in the regulation of T-ALL proliferation.

### Pharmacologic inhibition of USP7 induces apoptosis in T-ALL cells

**Fig. 6:**
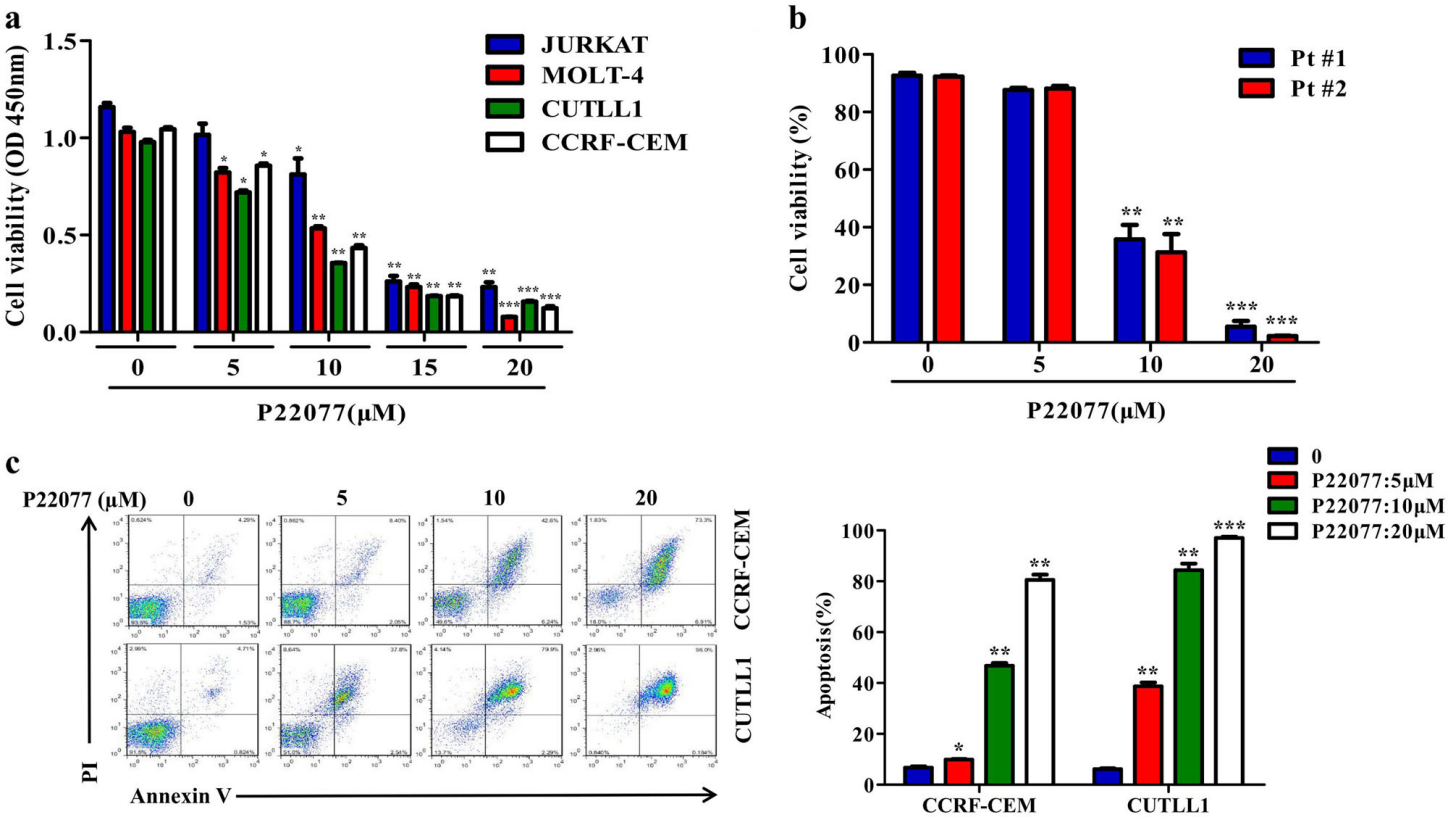
USP7 inhibition induces apoptosis in T-ALL cells. **a** T-ALL cell lines (JURKAT, MOLT-4, CUTLL1, and CCRF-CEM) were treated with increasing concentrations of P22077 for 24 h, followed by assessment of cell viability using a CCK-8 assay. The data are presented as the mean ± S.D. (Student’s *t*-test) of three independent experiments; ^*^p *<* 0.05, ^**^p *<* 0.01, and ^***^p < 0.001. **b** Primary T-ALL cells were isolated and treated with different concentrations of P22077 for 24 h, and the cell viability was monitored via trypan blue staining. The data are presented as the mean ± S.D. (Student’s *t*-test); ^**^p *<* 0.01, and ^***^p < 0.001. **c** CCRF-CEM and CUTLL1 cells were treated with various concentrations of P22077 for 48 h. Cell apoptosis was determined via flow cytometry using an Annexin-V/PI dual staining assay. Columns (right panel) represent the average percentage of Annexin V-positive cells from three independent experiments, shown as the mean ± S.D. (Student’s *t*-test); ^*^p *<* 0.05, ^**^p *<* 0.01, and ^***^p < 0.001

Encouraged by the observation that the knockdown of USP7 inhibited the proliferation of T-ALL cells, we next evaluated the effect of the USP7 inhibitor P22077 on cell viability in four T-ALL cell lines. The results showed that P22077 decreased the cell viability in a dose-dependent manner (Fig. 6a). More importantly, P22077 induced a dose-dependent cytotoxicity in primary T-ALL cells isolated from PBMCs of two T-ALL patients (Fig. 6b). Using an Annexin V/PI double-staining assay, we discovered that P22077 induced a remarkable level of apoptosis in CCRF-CEM and CUTLL1 cells (Fig. 6c). We also tested the anti-tumor effect of P22077 on other B-cell-derived hematologic malignant cells and found that T-ALL cells exhibited high sensitivity to P22077 treatment (Supplementary Fig. S2). These results indicate that USP7 inhibition by P22077 is cytotoxic to T-ALL cell lines and patient T-ALL cells.

## Discussion

The NOTCH1 signaling pathway enables direct transduction of extracellular signals at the cell membrane into transcriptional responses in the nucleus and plays a critical role during T cell development. It is also involved in the pathogenesis of over 60% of T-ALL cases.^6^ Accumulating evidence suggests that controlling the stability of NOTCH1 represents a novel approach to regulate the NOTCH1 signaling pathway and thus is attracting increasingly more attention. In the present study, for the first time, we identified USP7 as the deubiquitinase that maintains NOTCH1 stability and protects NOTCH1 from proteasome-mediated degradation. Therefore, targeting USP7 may represent a potential novel strategy to combat T-ALL.

The identification of frequent activating *NOTCH1* mutations in T-ALL has attracted significant interest in targeting the NOTCH1 signaling pathway for treatment.^11^ Several strategies have been undertaken to block NOTCH1 pathway signal transduction, including preventing ligand-triggered activation or NOTCH1 autoactivation by using monoclonal antibodies such as OMP-52M51, which binds to the negative regulatory domain of NOTCH1.^43^ In addition, γ-secretase inhibitors (GSIs) have been utilized to inhibit the γ-secretase-induced cleavage and release of the intracellular domain of NOTCH1 (ICN1). GSIs are the most studied potential agents targeting the NOTCH1 pathway. However, in previous studies, these inhibitors showed limited efficacy and intolerable side-effects.^7^ Recent studies revealed that proteasome inhibitors exert cytotoxicity and increase the chemosensitivity of T-ALL cells through suppression of NOTCH1 transcription.^44^ In addition, activation of the E3 ubiquitin ligase CHIP results in degradation of NOTCH1 and inhibits the proliferation of T-ALL cells.^15^ Based on the findings of these studies, we hypothesized that the inhibition of NOTCH1 deubiquitination catalysis processes might promote degradation of NOTCH1 and block the oncogenic NOTCH1 pathway. Here, we established that USP7 is responsible for NOTCH1 stabilization, which was supported by several lines of evidence. First, our results showed that USP7 interacted and was co-localized with ICN1 in T-ALL cells. Second, overexpression of USP7 removed ubiquitin from ICN1 in vivo and directly eliminated ubiquitin from purified ubiquitinated ICN1 in vitro. In contrast, USP7 inhibition increased ICN1 ubiquitination. Similar to its interaction with MDM2 or p53,^24^ both the N-terminal MATH domain and the C-terminal UBL domains of USP7 are needed for interaction with ICN1. The absence of either domain reverses USP7-mediated deubiquitination of ICN1. It is likely that ICN1 competes with MDM2 or p53 to bind USP7 in T-ALL. Finally, inhibition of the USP7 catalytic activity or depletion of USP7 reduced the protein level of ICN1 without affecting the mRNA level. Interestingly, USP7 inhibition caused degradation of ICN1 in T-ALL cell lines regardless of the status of NOTCH1 (JURKAT and CCRF-CEM cells have *FBW7* mutations; MOLT-4 and KOPT-K1 cells have PEST domain mutations;^45^ CUTLL1 cells harbor a chromosomal translocation at (7;9)(q34;q34)^46^). These findings indicate that other E3 ligases, such as CHIP, may be involved in the NOTCH1 ubiquitination catalysis process in T-ALLs with *FBW7* mutations or PEST domain mutations. Thus, these observations provide new insight into the regulation of the NOTCH1 deubiquitination processes in T-ALL cells.

USP7 has been found to be a drug target in several cancer types. However, its role in T-ALL is unclear. Similar to other cancers, USP7 is overexpressed in T-ALL, suggesting a possible role in T-ALL pathogenesis. In this regard, we discovered that knockdown of USP7 blocked T-ALL cell proliferation in vitro and in vivo, and inhibition of USP7 resulted in T-ALL cell growth suppression and apoptosis. Interestingly, USP7 inhibitor suppressed cell growth in both GSI-sensitive (CUTLL1) and GSI-resistant (JURKAT, MOLT-4, and CCRF-CEM) T-ALL cells, thus providing a rationale for inclusion of USP7 inhibitors in a GSI-based regimens for treatment of T-ALL patients. More importantly, we found that USP7 is overexpressed in human T-ALL, which likely results from the constitutive activation of NOTCH1. Bioinformatics analysis revealed that the *USP7* mRNA expression was higher in *NOTCH1* mutant than in the *NOTCH1* wild-type T-ALL patients, indicating that mutation of *NOTCH1* may upregulate the mRNA level of *USP7*. A positive feedback loop may exist between USP7 and NOTCH1: USP7 stabilizes and deubiquitinates NOTCH1, which in turn facilitates transactivation of *USP7*. Further studies are needed to verify this speculation.

Although the induction of NOTCH1 degradation may play a major role, we could not rule out the possibility that other USP7 substrates also contribute to the anti-leukemia effects upon USP7 inhibition. For example, PTEN has been shown to be involved in the pathogenesis of T-ALL.^47^ In an earlier study, USP7 removed PTEN monoubiquitination, enhanced its nuclear export, and abolished its nuclear function.^21^ MDM2 is also a substrate of USP7, and inhibition of USP7 resulted in degradation of MDM2. Nutlin-3a, an antagonist of MDM2, was reported to be able induce apoptosis in T-ALL cells with wild-type p53.^48^ These reports further support the notion that targeting USP7 is helpful in the treatment of T-ALL.

One concern for targeting USP7 in T-ALL emerges from recent observations of frequent loss-of-function mutations in *USP7* found in pediatric leukemia, especially in TAL1/LMO1-positive T-ALL cases.^38,49,50^ The role of USP7 in these patients remains enigmatic. Possibly, USP7 may function as a tumor suppressor. However, we cannot rule out the likelihood that USP7 may exert other catalytic-independent roles in these patients. These observations add to the complexity of the potential application of USP7-based therapy in T-ALL patients. Future studies are required to characterize the patients suitable for USP7-targeting therapy.

In summary, we identified USP7 as a novel deubiquitinase for NOTCH1 and found that targeting USP7 promoted NOTCH1 degradation, thus providing a novel means to terminate NOTCH1 signaling. Our findings provide a molecular basis and rationale for inclusion of USP7 inhibitors in T-ALL treatment strategies.

## Electronic supplementary material

supplemental material
