# Supplementary material for: USP7 deubiquitinates and stabilizes NOTCH1 in T-cell acute lymphoblastic leukemia
Source: Signal Transduct Target Ther. 2018 Oct 26;3:29. doi: 10.1038/s41392-018-0028-3 (PMC6202415; doi:10.1038/s41392-018-0028-3)

**USP7 deubiquitinates and stabilizes NOTCH1 in T-cell acute lymphoblastic leukemia**

Huizhuang Shan^1,5^, Xiangyun Li^1,5^, Xinhua Xiao^3,5^, Yuting Dai^3^, Jinyan Huang^3^, Junjun Song^4^, Meng Liu^1^, Li Yang^1^, Hu Lei^1^, Yin Tong^2^, Li Zhou^3^, Hanzhang Xu^1^, Yingli Wu^1*^

^*^Correspondence: [wuyingli@shsmu.edu.cn](mailto:wuyingli@shsmu.edu.cn)

**Supplementary Figure**

**Supplementary Figure S1.** (a-b) JURKAT and MOLT-4 cells were treated with increasing amounts of P22077 for 6 h as indicated. Total RNAs were collected for qRT-PCR analysis. Data were presented as means ± S.D. of three independent experiments, NS indicated no significant.


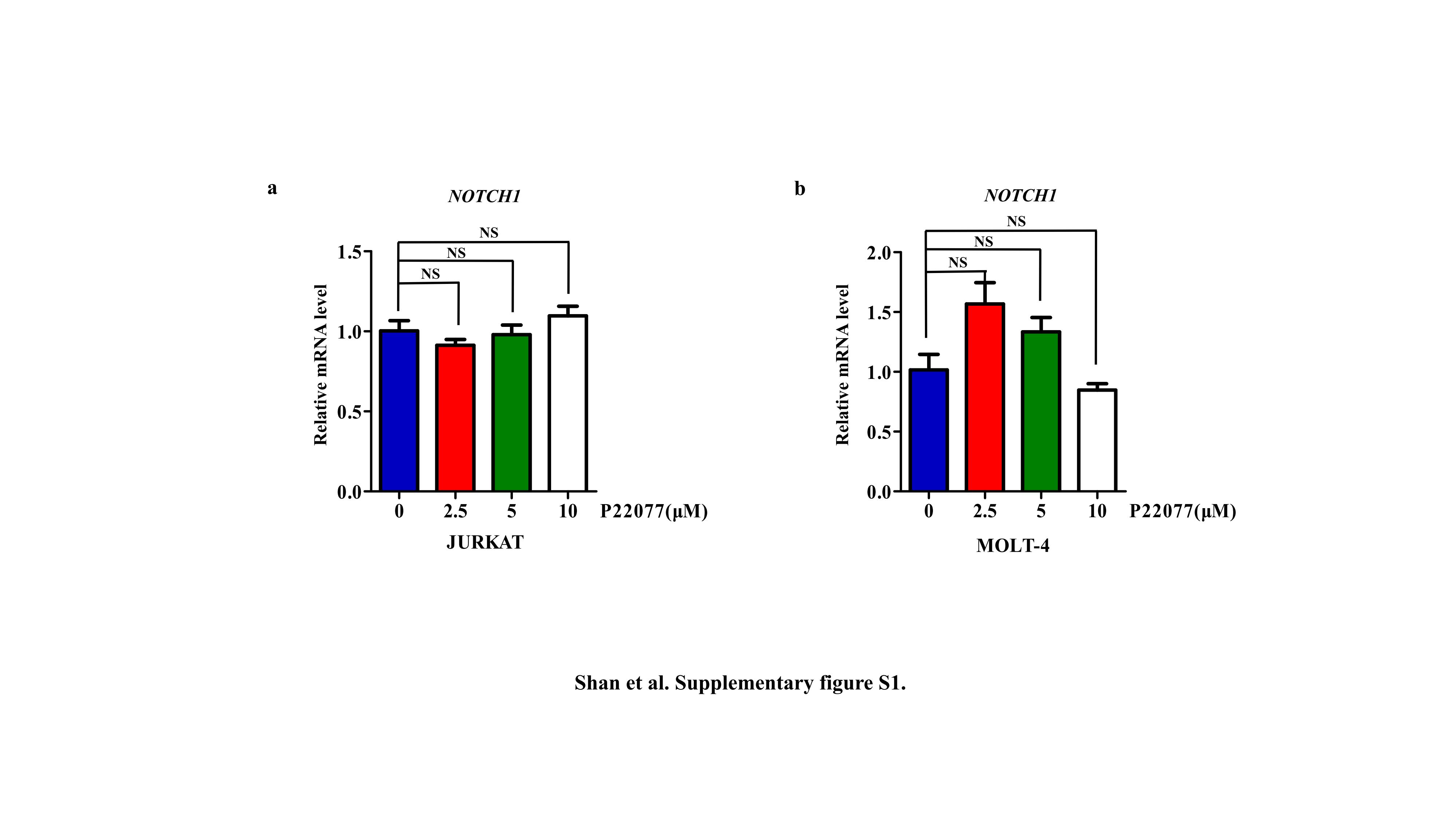


**Supplementary Figure S2.** Cells (T-ALL cell line: JURKAT; B-ALL cell line: SUP-B15; Multiple Myeloma cell lines: RPMI-8226 and ARP1; Burkitt's Lymphoma cell line: DAUDI) were treated with P22077 (10 μM and 15 μM) for 24 h, followed by assessment of cell viability using CCK-8 assay. Data are presented as means ± S.D.


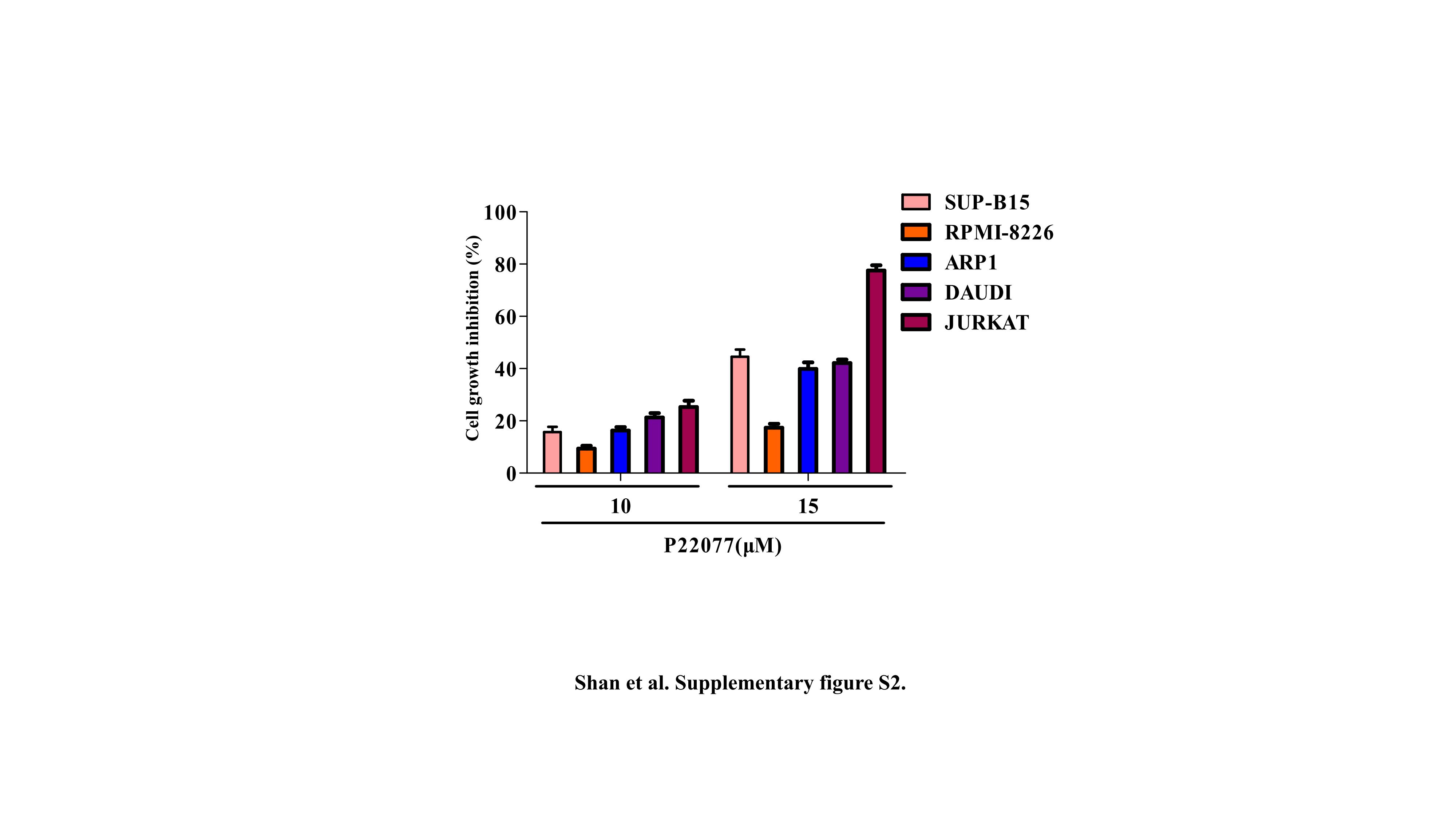

Supplement: Supplementary file 1 — supplemental material [file 41392_2018_28_MOESM1_ESM.docx]
